# Editorial: Rodent model organisms: therapeutic treatments and drugs interaction with the gut microbiome, volume II

**DOI:** 10.3389/fmicb.2026.1962495

**Published:** 2026-09-24

**Authors:** Julio Plaza-Diaz, Chrysoula Chrysa Voidarou, Eugenia Bezirtzoglou, Elisavet Stavropoulou

**Affiliations:** 1 Unitat de Nutrició Humana, Grup d'Alimentació, Nutrició, Desenvolupament i Salut Mental (ANUT-DSM), Departament de Bioquímica i Biotecnologia, Universitat Rovira i Virgili, Reus, Spain; 2 Institut de Recerca Biomèdica Catalunya Sud, Hospital Universitari Sant Joan de Reus, Reus, Spain; 3 Centro de Investigación Biomédica en Red de Fisiopatología de la Obesidad y Nutrición (CIBEROBN), Instituto de Salud Carlos III (ISCIII), Madrid, Spain; 4 Laboratory of Animal Health, Food Hygiene and Quality, Department of Agriculture, School of Agriculture, University of Ioannina, Arta, Greece; 5 Laboratory of Hygiene and Environmental Protection, Department of Medicine, Democritus University of Thrace, Alexandroupolis, Greece; 6 University of Nicosia-UNIC Athens, Athens, Greece; 7 Infectious Diseases Service, Department of Medicine, Lausanne University Hospital, University of Lausanne, Lausanne, Switzerland

**Keywords:** fecal microbiota transplantation, gut microbiome, pharmacomicrobiomics, probiotics, rodent models, therapeutic interventions

## 1 Opening: the gut microbiome as a therapeutic interface

The gut microbiome has emerged as a central regulator of host physiology, extending its influence far beyond the intestinal environment. Composed of diverse microbial communities that interact continuously with host tissues, the gut microbiome contributes to essential biological processes, including nutrient metabolism, immune system regulation, maintenance of intestinal barrier integrity, and communication with extraintestinal organs through microbial metabolites and signaling molecules. Increasing evidence indicates that disruptions in microbial composition and function are associated with a wide range of pathological conditions affecting the gastrointestinal tract as well as distant systems, including the liver, brain, cardiovascular system, lung, kidney, endocrine organs, and immune system.

This expanding understanding has transformed the microbiome from a passive indicator of disease status into an active therapeutic interface. Pharmacological treatments, dietary interventions, traditional medicines, natural products, and complementary therapies can reshape microbial communities by altering microbial abundance, diversity, metabolic activity, and ecological interactions. These changes may contribute to therapeutic outcomes by modifying inflammation, oxidative stress, immune responses, and host metabolic pathways. However, the relationship is not unidirectional. Microorganisms and their metabolites can influence the absorption, metabolism, bioavailability, efficacy, and toxicity of therapeutic agents. Microbial enzymes may transform drugs into active or inactive metabolites, while microbiota-derived molecules such as short-chain fatty acids, bile-acid derivatives, and tryptophan metabolites can regulate host signaling pathways that determine treatment responses.

Therefore, the interaction between therapeutics and the gut microbiome represents a dynamic and bidirectional relationship: treatments can act upon microbial ecosystems, while microbial communities can modify the biological effects of treatments. This concept has given rise to the expanding field of pharmacomicrobiomics, which seeks to understand how microbial variation contributes to differences in therapeutic efficacy and adverse effects. Such knowledge is particularly relevant for complex interventions, including traditional herbal formulations and microbiota-targeted therapies, where multiple active components may interact with microbial networks and host pathways simultaneously.

Controlled animal models remain indispensable for investigating these mechanisms. Rodent models allow precise manipulation of microbiota composition, dietary conditions, disease states, and therapeutic exposures while enabling repeated sampling of tissues, microbiological analyses, and molecular pathway validation. These experimental systems provide causal insights that are difficult to obtain in human studies, particularly when invasive procedures, longitudinal sampling, or controlled environmental conditions are required. By integrating microbiology, pharmacology, immunology, and systems biology, animal models continue to provide a critical foundation for understanding how the gut microbiome influences therapeutic responses and for developing microbiome-informed strategies for disease prevention and treatment.

## 2 Objectives and scope of volume II

Building on the first volume of this Research Topic, Volume II was conceived to deepen understanding of the reciprocal interactions between therapeutic interventions, pharmacological agents, and the gut microbiome in experimentally controlled animal models. Its central objective was not only to document treatment-associated shifts in microbial composition, but also to clarify whether these changes participate in therapeutic efficacy, toxicity, drug disposition, and disease modification. The Research Topic therefore encompasses conventional medicines, traditional formulations, botanical and natural bioactive compounds, complementary physical interventions, probiotics, commensal microorganisms, fecal microbiota transplantation, functional foods, and defined microbial metabolites (Bezirtzoglou et al.).

Across its 46 contributions, the Research Topic covers a broad spectrum of gastrointestinal and extraintestinal disorders, including inflammatory bowel disease, metabolic and cardiovascular disease, liver and kidney injury, neurological and behavioral conditions, pulmonary disorders, cancer, reproductive dysfunction, and treatment-induced tissue injury. Collectively, these studies extend the field beyond descriptive taxonomic profiling by integrating microbiome analyses with metabolomics, pharmacokinetics, histopathology, intestinal barrier assessment, immune phenotyping, and pathway-specific validation. They also highlight recurrent mechanisms involving microbial metabolites, epithelial integrity, inflammatory signaling, oxidative stress, and communication along gut–organ axes. The contributions can consequently be synthesized into five interrelated themes: pharmacological treatments and treatment-associated perturbations; traditional, natural, and complementary interventions; direct microbiota-targeted therapies; gastrointestinal inflammation and barrier dysfunction; and extraintestinal microbiome-mediated mechanisms.

## 3 Conventional pharmacological treatments and treatment-associated microbiome perturbations

The relationship between pharmacological treatments and the gut microbiome is bidirectional. Therapeutic agents can alter microbial community structure, microbial metabolic activity, and intestinal barrier function, potentially contributing to their beneficial or adverse effects. Conversely, the composition and functional capacity of the microbiome may influence drug metabolism, systemic exposure, treatment efficacy, and host physiological responses. The studies grouped in this section illustrate these interactions across antidiabetic therapies, experimental microbiota depletion, pharmacokinetic variability, and treatment-associated tissue injury.

### 3.1 Drug efficacy associated with microbiome modulation

Several contributions investigated whether established treatments produce effects extending beyond their canonical pharmacological targets. In mice with diabetic kidney disease, He J. et al. showed that empagliflozin improved renal outcomes while modifying gut microbial diversity and community composition.

Notably, the baseline microbiota, treatment-associated microbial responses, and correlations between selected taxa and renal or metabolic parameters differed between male and female mice. These results identify biological sex as a potentially important source of heterogeneity in microbiome-associated therapeutic responses. However, because the microbial findings were primarily correlational, additional experiments are needed to determine whether these changes contributed directly to renoprotection or accompanied improvements in the underlying disease.

Similarly, Chang et al. reported that sitagliptin partially restored microbial diversity and shifted the microbial profile of diabetic rats toward that observed in healthy controls. Treatment also modified predicted microbial functions, promoted short-chain fatty acid-related activity, increased the expression of the tight-junction protein zonula occludens-1 (ZO-1), and increased the abundance of mucin-secreting goblet cells. These findings connect antidiabetic treatment with a broader microbiota–metabolism-intestinal barrier network, although they do not establish whether microbial restoration is a mediator of glycemic improvement or a secondary response to improved metabolic status.

A complementary tissue-centered perspective was provided by Silva et al., who evaluated exogenous melatonin in rats with domperidone-induced hyperprolactinemia. Microscopic, histomorphometric, immunohistochemical, and computational analyses identified structural and proliferative alterations in the stomach, duodenum, and liver. Melatonin modulated body weight and selected duodenal parameters but did not consistently reverse the gastrohepatic alterations associated with hyperprolactinemia. Although this study did not assess the gut microbiome directly, it broadens the Research Topic methodologically by demonstrating the value of organ-level histological and computational analyses when evaluating systemic treatment responses.

### 3.2 Microbiome effects on pharmacokinetics and systemic physiology

The reverse influence of the microbiome on pharmacological responses was demonstrated more directly by Xia et al.. Using models of endogenous and ceftriaxone-induced dysbiosis, the authors showed that microbial disruption reduced the oral bioavailability of losartan and its active metabolite.

The abundance of *Enterococcus faecalis* was inversely associated with pharmacokinetic measures of losartan exposure, while complementary *in vitro* experiments showed that this bacterium could degrade losartan and convert it into E-3179. This study therefore provides direct mechanistic support for pharmacomicrobiomics by showing that specific microbial alterations can contribute to variability in drug disposition.

Microbiota-dependent changes in systemic physiology were also examined by Kellenberger et al.. Antibiotic-induced microbiota depletion improved glucose tolerance and increased circulating concentrations of active glucagon-like peptide-1 (GLP-1). The improvement in glucose tolerance was absent in mice lacking the GLP-1 receptor, supporting the involvement of GLP-1 signaling. Bile acid profiling and complementary validation experiments further identified potential inhibitors of basal GLP-1 secretion. These findings demonstrate that microbiota depletion is not a biologically neutral manipulation, but one capable of altering enteroendocrine signaling and systemic glucose regulation.

### 3.3 Treatment-associated microbial and metabolic injury

Therapeutic exposures may also produce adverse effects through coordinated microbial, metabolic, inflammatory, and tissue-level disturbances. Lu et al. characterized the acute consequences of total abdominal irradiation in mice. Irradiation produced progressive intestinal injury, increased permeability, reduced microbial diversity, depleted potentially beneficial and butyrate-producing bacteria, and increased potentially harmful taxa.

These alterations were accompanied by changes in microbial metabolites and activation of the Toll-like receptor 4 (TLR4)/myeloid differentiation primary response 88 (MyD88)/nuclear factor kappa B (NF-κB) inflammatory pathway. This integrated analysis illustrates how a therapeutic exposure can simultaneously disrupt the microbial ecosystem, metabolome, epithelial barrier, and inflammatory signaling network.

Collectively, these studies show that drug–microbiome interactions encompass therapeutic efficacy, host heterogeneity, pharmacokinetics, enteroendocrine regulation, and treatment-associated toxicity. Microbial changes observed after treatment may function as causal mediators, modifiers of therapeutic response, compensatory adaptations, or secondary consequences of disease improvement. Distinguishing among these possibilities will require microbial transfer, depletion, and recolonization experiments, together with targeted validation of candidate microorganisms, metabolites, and host signaling pathways.

## 4 Traditional medicines, natural products and complementary systemic interventions

A prominent theme emerging from this Research Topic is the capacity of traditional medicines, plant-derived bioactive compounds, and complementary non-pharmacological interventions to influence disease progression through interactions with the gut microbiome. Unlike conventional drug discovery approaches that frequently target a single molecular pathway, many of these interventions appear to act through interconnected networks involving microbial ecology, host metabolism, immune regulation, oxidative stress responses, and neuroendocrine signaling. Rodent and other experimental animal models remain particularly valuable for dissecting these relationships because they permit controlled manipulation of microbiota composition, dietary exposures, disease induction protocols, and tissue-specific molecular analyses that are often not feasible in human studies.

### 4.1 Cardiometabolic, renal, and hepatic interventions

Several studies within this Research Topic demonstrate that microbiome-associated mechanisms contribute substantially to the therapeutic effects of natural compounds in cardiometabolic and liver disorders. In a rabbit model of atherosclerosis, Liu F.-Y. et al. examined how a marine-derived polysaccharide interacted with conventional lipid-lowering therapy. The study showed that fucoidan, either alone or combined with simvastatin, was associated with improvements in atherosclerotic pathology accompanied by alterations in gut microbial communities and metabolite profiles. These findings illustrate the increasingly recognized concept that therapeutic efficacy in cardiovascular disease may arise not only from direct host-targeted effects but also from microbiota-mediated metabolic remodeling.

Within metabolic and renal disorders, Huang, Wang et al. investigated the anti-hyperuricemic effects of mulberry leaf and identified kaempferol as its principal candidate bioactive compound. By combining network pharmacology, molecular docking, molecular-dynamics simulations, and experimental validation in a mouse model of hyperuricemia, the authors identified AKT1 and TNF as relevant molecular targets. *In vivo*, kaempferol reduced uric acid concentrations, modulated purine metabolism, attenuated renal oxidative stress and inflammatory responses, and decreased AKT expression and phosphorylation. Treatment also partially corrected the gut microbial dysbiosis associated with hyperuricemia. This study therefore connects a defined plant-derived compound with molecular signaling, renal protection, and microbial regulation, supporting the gut–kidney axis as a potential component of its therapeutic effects. Nevertheless, additional microbial-transfer or depletion experiments would be required to establish whether restoration of the microbiota directly mediates the anti-hyperuricemic response.

The importance of microbial homeostasis in metabolic liver disease was further highlighted by Wang X.-L. et al.. By using a model of non-alcoholic fatty liver disease, the authors demonstrated that paeoniflorin reduced hepatic injury while simultaneously attenuating oxidative stress and inflammatory responses. Restoration of microbial balance appeared closely linked to these protective effects, supporting the view that intestinal dysbiosis contributes to disease progression and that microbial normalization may represent a key therapeutic mechanism in chronic liver disorders.

Similarly, Li K. et al. connected microbial modulation with activation of antioxidant defense pathways. Hesperidin treatment ameliorated acute liver injury while influencing gut microbial composition and regulating the nuclear factor erythroid 2-related factor 2 (Nrf2)/Kelch-like ECH-associated protein 1 (Keap1) signaling axis, a central cellular defense mechanism against oxidative damage. The study provides an important example of how microbiome-associated interventions may converge on established molecular pathways to produce hepatoprotective effects.

The relationship between microbiota regulation and protection from drug-induced toxicity was explored by Wang D. et al.. Anti-tuberculosis therapies are frequently associated with hepatotoxicity, representing a major clinical challenge. In this model, wolfberry supplementation mitigated liver injury while modulating gut microbial communities and influencing Yes-associated protein 1 (YAP1)/farnesoid X receptor (FXR)-related signaling pathways involved in bile acid metabolism and hepatic homeostasis. These findings highlight the possibility that microbiota-directed strategies may serve as adjunctive approaches for reducing adverse effects associated with long-term pharmacological treatments.

Collectively, these studies demonstrate that natural products can exert systemic benefits extending beyond direct pharmacological activity. By reshaping microbial ecosystems and their metabolic outputs, these interventions influence inflammatory processes, oxidative stress responses, lipid metabolism, and liver function, emphasizing the microbiome as an important mediator of cardiometabolic, renal, and hepatic health.

### 4.2 Neurobehavioral disorders, frailty and neurodegeneration

The microbiota–gut–brain axis has emerged as a major focus of contemporary biomedical research, and several contributions in this Research Topic extend this concept to frailty, neurodegenerative disease, insomnia, and depression. Modulation of intestinal microbial communities was associated with improvements in physiological frailty in a transgenic Alzheimer's disease (AD) model, as reported by Yu T. et al.. These findings reinforce growing evidence that age-related decline and neurodegenerative processes may be influenced by microbiome-dependent mechanisms that extend beyond the central nervous system.

A complementary perspective was provided by Deng et al., who compared responses across different mouse strains and disease states. By integrating microbiome and metabolomic analyses, the study illustrated how electroacupuncture-induced benefits may involve coordinated alterations in microbial composition and host metabolic pathways. The comparative design further emphasized the value of animal models for identifying disease-specific and host-specific responses to microbiota-targeted interventions.

Sleep disorders were addressed by Wang Q. et al., who demonstrated that a traditional botanical formulation improved insomnia-related outcomes through mechanisms involving gut–brain communication. The work supports the hypothesis that microbial metabolites and neuroactive signaling pathways may contribute to sleep regulation and therapeutic responses.

Notably, microbiome-associated benefits were not restricted to pharmacological or botanical interventions. Liu J. et al. demonstrated that a physical therapeutic approach could influence both gut microbial composition and brain–gut peptide signaling. These findings suggest that non-drug interventions may also engage microbiota-dependent pathways relevant to neurological and behavioral health.

Finally, Li X. et al. highlighted the interconnected nature of psychological and gastrointestinal symptoms. Improvements in depressive-like behaviors occurred alongside correction of constipation and restoration of microbial balance, further supporting the concept that behavioral and gastrointestinal outcomes may be linked through shared microbiota-mediated mechanisms.

Taken together, these studies illustrate the breadth of microbiome-associated therapeutic strategies currently being explored in animal models. From herbal medicines and natural compounds to electroacupuncture and physical therapies, diverse interventions appear capable of modifying microbial ecosystems and, through them, influencing systemic physiology, neurobehavioral function, and disease progression. Such findings underscore the importance of rodent and related experimental models for unraveling the causal mechanisms that connect complementary therapies, host biology, and the gut microbiome.

### 4.3 Pulmonary disease and cancer

Beyond cardiometabolic, hepatic, and neurobehavioral disorders, the studies in this Research Topic further demonstrate that microbiome-mediated therapeutic effects extend to cancer and pulmonary diseases. These investigations reinforce the concept that the gut microbiome functions as a systemic regulatory network capable of influencing distant organs through microbial metabolites, immune signaling pathways, and host metabolic responses. Importantly, the integration of microbiome profiling with metabolomics has emerged as a powerful approach for identifying mechanistic links between traditional medicines and disease outcomes.

The growing application of multi-omics methodologies is exemplified by Li P. et al.. Using a tumor-bearing mouse model, the study combined gut microbial analyses with metabolomic profiling to characterize the antitumor actions of Jinfu'an decoction. Rather than attributing efficacy to a single molecular target, the findings suggest that therapeutic effects arise from coordinated changes in microbial composition and host metabolic pathways. Such studies illustrate how microbiome–metabolome interactions may influence tumor growth through modulation of immune responses, metabolic homeostasis, and systemic inflammatory processes. They also highlight the increasing importance of system-level approaches for understanding the complex mechanisms underlying traditional herbal therapies.

Several contributions further expanded the scope of microbiome research to pulmonary diseases, emphasizing the importance of the gut–lung axis. In the study by Chen Y.-x. et al., administration of Huanglong Zhixiao Formula promoted the restoration of gut microbial homeostasis and enhanced immune regulation in a murine model of asthma, highlighting the potential role of microbiota-mediated mechanisms in modulating airway inflammatory responses. These findings support the growing recognition that intestinal microorganisms can influence pulmonary immunity through circulating metabolites, immune cell trafficking, and inflammatory mediators. The study provides additional evidence that microbiota-targeted interventions may represent a valuable strategy for modulating chronic airway inflammation.

The gut–lung axis was further investigated by Sun et al. in a model of idiopathic pulmonary fibrosis, demonstrating that modulation of gut microbiota composition and metabolic profiles may contribute to the regulation of pulmonary disease progression. By linking microbial regulation to systemic metabolic remodeling, the study broadens our understanding of how intestinal ecosystems may influence chronic fibrotic processes occurring in distant organs.

An additional dimension of gut–lung communication was investigated by He Y. et al., who demonstrated that regulation of intestinal microbiota and microbial metabolites contributed to the amelioration of hypoxic pulmonary hypertension, further expanding the role of microbiome-mediated mechanisms in pulmonary vascular disorders. These findings suggest that microbiome-derived metabolic signals may contribute not only to pulmonary inflammation but also to vascular adaptation and cardiopulmonary homeostasis under hypoxic conditions. The work extends the relevance of microbiome-centered therapeutic strategies to pulmonary hypertension and related vascular disorders.

Taken together, these studies reveal remarkable convergence despite the diversity of diseases and interventions examined. Whether addressing atherosclerosis, hyperuricemia and renal injury, fatty liver disease, drug-induced hepatotoxicity, frailty, neurodegeneration, insomnia, depression, cancer, asthma, pulmonary fibrosis, or pulmonary hypertension, many therapeutic benefits appear to involve common biological themes. These include restoration of microbial homeostasis, modulation of microbial metabolites, improvement of intestinal barrier integrity, attenuation of oxidative stress, and regulation of immune and inflammatory signaling pathways. At the same time, important challenges remain. Many traditional medicines contain multiple bioactive constituents whose individual contributions are difficult to define, while issues of dose standardization, formulation variability, and reproducibility across laboratories continue to complicate translation. Nevertheless, the consistent observation that diverse therapeutic modalities converge on microbiome-associated mechanisms underscores the value of experimental animal models for elucidating causal pathways and identifying novel microbiota-targeted strategies for systemic disease management.

## 5 Direct microbiota-targeted therapies and microbial metabolites

While many studies in this Research Topic investigate therapies that influence the gut microbiome indirectly through pharmacological, nutritional, or complementary interventions, a distinct group of contributions focuses on strategies that directly manipulate microbial communities or administer microbial products. These approaches provide particularly valuable mechanistic insights because they target the microbiome itself rather than treating it as a secondary mediator of therapeutic effects. Collectively, the studies span three interconnected levels of intervention: restoration of entire microbial ecosystems through fecal microbiota transplantation (FMT), administration of selected probiotic or commensal microorganisms, and supplementation with defined microbial metabolites. Together, they illustrate an evolving continuum from community-level microbial engineering to increasingly precise microbiota-derived therapeutics.

### 5.1 Fecal microbiota transplantation and community restoration

FMT represents one of the most comprehensive microbiota-directed therapeutic strategies, as it aims to re-establish intestinal ecological balance through the transfer of an entire microbial community. This ecosystem-level intervention was investigated by Luo et al., who evaluated the combined effects of FMT and the bioactive natural compound andrographolide in an experimental model of colitis. This study demonstrated that microbial community restoration and host-directed pharmacological intervention can act synergistically, suggesting that therapeutic success may depend on both re-establishing microbial functions and modifying host inflammatory responses. Such findings support the concept that microbiome restoration may enhance the efficacy of complementary therapeutic agents.

The therapeutic consequences of FMT were shown to depend strongly on disease context and biological sex in the study by Soriano et al.. The authors administered sex-matched FMT from healthy C57BL/6 donors to male and female 5xFAD mice after traumatic brain injury and assessed amyloid pathology, glial activation, microbial composition, short-chain fatty acids, intestinal histology, and motor performance. Traumatic brain injury intensified amyloid deposition, neuroinflammation, and microbial disruption, with female mice showing greater vulnerability in several outcomes. FMT modified microbial community patterns and reduced amyloid deposition in uninjured animals, while its effects on glial activation differed according to sex and brain region. However, acute FMT did not reverse the increase in amyloid pathology, neuroinflammation, or motor impairment produced by traumatic brain injury. These findings provide an important counterpoint to uniformly beneficial interpretations of microbiota transplantation, indicating that its effects may depend on sex, neurological comorbidity, intervention timing, and the severity of the underlying pathological disturbance.

A broader historical and conceptual perspective on microbiota-based therapeutic approaches was presented by Li M. et al.. Linking traditional medical practices with contemporary microbiota-transfer strategies, this work highlights the long-standing recognition that manipulation of intestinal contents can influence health and disease. The review emphasizes how modern sequencing technologies and mechanistic studies have transformed empirical observations into a scientifically grounded field of microbiota-based medicine.

Li D. et al. further investigated the immunological effects of microbiota restoration, demonstrating that both microbial communities and their derived metabolites contributed to disease improvement through modulation of the Th17/Treg balance, a key regulator of intestinal immune homeostasis. These findings emphasize that effective microbiota-based therapies depend not only on the reintroduction of microorganisms but also on the restoration of microbial functional capacity and metabolite-driven regulation of host immune responses.

### 5.2 Probiotics and candidate commensal therapeutics

Compared with FMT, probiotic and commensal-based approaches offer greater standardization and control by introducing selected microbial strains with defined biological properties. Several studies in this Research Topic illustrate the expanding diversity of candidate live biotherapeutics and their mechanisms of action.

In the study by Lin Y. et al., probiotic supplementation mitigated toxin-induced damage by concurrently attenuating oxidative stress, reducing inflammatory responses, and re-establishing microbial balance. These findings highlight the potential of specific probiotic strains as protective biological interventions against environmental and food-derived toxic challenges.

Similarly, Chen H. et al. demonstrated the therapeutic potential of a candidate commensal microorganism in an infectious disease model. Through modulation of microbial community structure and reduction of disease severity, *Parabacteroides distasonis* represents a promising example of next-generation microbiota-based therapeutics that extend beyond conventional probiotic approaches.

The anti-inflammatory potential of probiotic interventions was further demonstrated by Fu et al., who reported improvements in experimental colitis associated with alterations in microbial composition and immune responses. Alongside findings from FMT-based approaches, these results further support the pivotal role of microbiota modulation in regulating inflammatory bowel disease progression and recovery.

Importantly, the effects of probiotic interventions extend beyond gastrointestinal disorders. Chen M. et al. demonstrated that microbiota-targeted strategies can modulate systemic immune function by enhancing vaccine-induced immune responses, highlighting the broader immunological impact of probiotic-based therapies. Similarly, Lin J. et al. expanded the application of microbiome research to diabetic microvascular complications, demonstrating that regulation of intestinal microbial communities may influence retinal disease through mechanisms involving the gut–retinal axis.

The growing sophistication of live biotherapeutic development is exemplified by Yu Y. et al.. Rather than focusing solely on microbial abundance, the study linked administration of a defined commensal species to alterations in tryptophan metabolism, aryl hydrocarbon receptor (AhR) signaling, and behavioral outcomes. Such work illustrates how candidate microbial therapeutics may influence neurodevelopmental and neurobehavioral disorders through highly specific host–microbe signaling pathways.

### 5.3 Microbial metabolites as interventions

The most reductionist microbiota-targeted strategies involve administration of defined microbial metabolites, allowing investigators to isolate functional molecules from the complexity of living microbial communities. This approach was illustrated by Wang Y. et al., which identified a microbiota-derived lipid mediator capable of reducing neuroinflammation through epigenetic and neurotrophic signaling mechanisms. By linking EPA to H3K9 acetylation and BDNF/TrkB signaling, the study provides a compelling example of how microbial metabolites can directly influence host cellular pathways relevant to neurological disease.

Taken together, these studies illustrate progression from ecosystem-level restoration through FMT, to administration of selected probiotic and commensal organisms, and finally to delivery of a purified microbial product. They also highlight an important methodological consideration for future microbiome research: stronger causal inference requires experimental designs capable of distinguishing the effects of transferred microorganisms, community-level ecological functions, and co-transferred metabolites. Rodent models remain uniquely valuable for achieving this level of mechanistic resolution, providing a foundation for the development of increasingly precise microbiota-based therapeutics.

## 6 Gastrointestinal inflammation, intestinal barrier dysfunction and functional bowel disorders

Disorders of the gastrointestinal tract represent some of the clearest examples of bidirectional interactions between the host and the gut microbiome. Across conditions such as diarrhea, irritable bowel syndrome (IBS), constipation, and ulcerative colitis, a recurring mechanistic sequence emerges disruption of microbial homeostasis leads to altered production of microbial metabolites and bioactive products, which subsequently influence host signaling pathways, compromise epithelial and mucus barrier integrity, and trigger immune activation. These events can ultimately affect intestinal motility, visceral sensitivity, inflammatory responses, and clinical symptoms. The studies collected in this Research Topic collectively support a disease-centered framework in which microbial dysbiosis functions as both a driver and a therapeutic target of gastrointestinal pathology.

### 6.1 Diarrhea and irritable bowel syndrome

Several investigations focused on diarrhea and diarrhea-predominant irritable bowel syndrome (IBS-D), highlighting the interconnected roles of microbial ecology, barrier integrity, and inflammatory signaling. In Zhao et al., a food-derived intervention improved diarrhea while simultaneously modulating gut microbial composition and enhancing intestinal barrier function. The findings support the concept that dietary components can influence gastrointestinal health not only through nutritional effects but also by reshaping microbial communities and preserving epithelial integrity.

The role of intestinal barrier dysfunction as a consequence of microbiota imbalance was further highlighted by Li H. et al.. In an experimental model of IBS-D, abdominal massage altered gut microbial composition and suppressed the lipopolysaccharide (LPS)/TLR4/NF-κB/myosin light-chain kinase (MLCK) signaling pathway. These changes were accompanied by improvements in intestinal barrier function and symptom severity, supporting a link between microbial dysbiosis, inflammatory signaling, and epithelial permeability.

A complementary mechanistic pathway was reported by Xie X. et al., emphasizing the contribution of microbiota-mediated signaling in gastrointestinal disease regulation. Rather than emphasizing inflammatory mediators alone, this work highlighted the role of microbiota-derived tryptophan metabolites in regulating the AhR, an important mediator of mucosal homeostasis and immune function. The findings reinforce the growing recognition that microbial metabolites act as signaling molecules connecting intestinal microorganisms with host epithelial and immune responses.

A comparative evaluation of therapeutic approaches was presented by Zhang et al.. By evaluating complementary and conventional treatments within the same experimental framework, the study demonstrated that distinct interventions could produce overlapping clinical improvements while exerting different effects on microbial community structure and microbial interaction networks. Such observations suggest that multiple therapeutic routes may converge on common physiological outcomes through partially distinct microbiome-associated mechanisms.

### 6.2 Constipation and gastrointestinal motility

Microbiome-associated mechanisms also play an important role in regulating intestinal motility disorders. Du et al. employed integrated metagenomic and metabolomic approaches to investigate the mechanisms underlying slow-transit constipation, revealing potential links between microbial alterations, metabolic changes, and impaired bowel function. The study demonstrated that alterations in microbial composition were closely associated with changes in metabolic pathways relevant to gastrointestinal motility. By combining two complementary omics approaches, the authors provided evidence that microbial metabolism may influence intestinal transit through complex host–microbe interactions rather than through isolated signaling pathways. These findings highlight the value of systems-level analyses for understanding the microbiological determinants of bowel function.

### 6.3 Ulcerative colitis, inflammatory bowel disease, and mucosal-barrier restoration

The largest group of gastrointestinal studies focused on ulcerative colitis and inflammatory bowel disease, conditions characterized by chronic inflammation, epithelial injury, and disruption of intestinal barrier integrity. A recurring theme across these investigations was the restoration of barrier function through coordinated modulation of microbial communities and microbial metabolites.

In the study by Liu D. et al., therapeutic effects were linked to the concurrent restoration of microbial balance, regulation of microbiota-derived metabolites, and improvement of intestinal epithelial barrier integrity. These findings highlight the intimate connection between microbial metabolic functions and processes involved in mucosal repair and recovery.

The contribution of specific microbial taxa to disease progression was investigated by She et al.. By using a defined pathobiont to enhance disease severity, the study provided a valuable experimental model for examining causal interactions between the microbiota and host responses. The observed recovery of intestinal barrier function following treatment further supports the close relationship between microbial composition and epithelial integrity as key determinants of disease progression and therapeutic outcome.

Microbial metabolic pathways and immune regulation were shown to intersect in the study by Jin et al. The authors demonstrated that changes in tryptophan metabolism influenced AhR signaling and the distribution of innate lymphoid cell populations, establishing a direct link between microbiota-derived metabolites and host immune modulation. These findings extend observations from IBS-D models by highlighting the broader role of microbial tryptophan metabolites in regulating both epithelial homeostasis and immune responses across diverse gastrointestinal disorders.

Further support for the interconnected relationship between the gut microbiota and intestinal barrier function was provided by Wu and Mao and Xu et al. Both studies demonstrated that microbial modulation was accompanied by reduced inflammatory signaling, improvements in cytokine profiles, enhancement of tight-junction integrity, and broader metabolic changes. These findings reinforce the concept that successful treatment of inflammatory bowel disease often requires simultaneous regulation of microbial ecology, epithelial barrier function, and host immune responses.

Taken together, the studies in this section support a unifying mechanistic framework in which dysbiosis initiates alterations in microbial metabolites and microbial products, including LPS and tryptophan-derived compounds. These changes subsequently influence signaling pathways such as TLR4 and AhR, contributing to epithelial and mucus-barrier disruption, immune activation, altered motility, and gastrointestinal dysfunction. Although the interventions investigated differ substantially in composition and therapeutic modality, their beneficial effects repeatedly converge on restoration of microbial homeostasis and barrier integrity, underscoring the central role of the gut microbiome in gastrointestinal health and disease.

## 7 Extraintestinal gut–organ axes and causal microbiome mechanisms

### 7.1 Cardiovascular and metabolic mechanisms

The contributions to this Research Topic demonstrate that microbial effects are not restricted to the gastrointestinal tract. Instead, microorganisms originating from different anatomical niches, together with their enzymatic functions and metabolites, can influence systemic metabolic pathways. Xie W. et al. investigated this relationship through an oral–gut metabolic axis involving *Porphyromonas gingivalis*. Oral exposure to this periodontal pathogen increased circulating trimethylamine-N-oxide (TMAO) under both standard and high-choline dietary conditions, while also altering gut microbial composition and reducing the expression of intestinal barrier proteins. Under high-choline conditions, *P. gingivalis* further increased cecal trimethylamine and the microbial choline–trimethylamine lyase markers *cutC* and *cutD*. These findings connect an oral pathogen with the functional capacity of the intestinal microbiome to metabolize dietary choline, providing a mechanistic link among periodontal exposure, gut microbial metabolism, and systemic trimethylamine-N-oxide accumulation.

A complementary example of microbiome-dependent metabolic regulation was provided by Duan et al. In mice with high-fat diet-induced obesity, low-dose oral nicotine attenuated weight gain, fat accumulation, hepatic steatosis, inflammation, and metabolic dysfunction. Microbiota depletion and fecal microbiota transplantation experiments supported the dependence of these effects on the intestinal microbial ecosystem. Metagenomic analyses identified enrichment of *Adlercreutzia*, and administration of this commensal reproduced several of the anti-obesogenic effects observed after nicotine exposure. Integrated metabolomic analyses further connected *Adlercreutzia* with changes in polyunsaturated fatty acid metabolism. Although nicotine itself cannot be interpreted as a general therapeutic recommendation, this experimental model illustrates how a pharmacological exposure can produce a systemic metabolic phenotype through a specific microorganism–metabolite pathway.

### 7.2 Enterohepatic and endocrine communication

Bile acids emerged as another important interface between the microbiome and systemic metabolism. Zou et al. examined bile acid profiles and intestinal microbial composition in a rat model of sustained hyperuricemia. Hyperuricemia was associated with increased hepatic cholic acid synthesis, accumulation of 12α-hydroxy bile acids in ileal contents, and changes in microbial community structure. The abundances of *Allobaculum* and *Bifidobacterium* were positively correlated with several 12α-hydroxy bile acids, including taurocholic, taurodeoxycholic, and glycocholic acids.

The study therefore identifies a potential connection among purine metabolism, hepatic bile acid synthesis, enterohepatic circulation, and the gut microbiome. Nevertheless, because the microbial and bile acid findings were primarily correlational, functional transfer or depletion experiments are still required to determine the direction of this relationship.

Stronger evidence for a microbiome-mediated endocrine phenotype was reported by Huang, Zhang et al.. Transplantation of gut microbiota from patients with gestational diabetes mellitus into antibiotic-treated pregnant mice induced glucose intolerance and reorganized the recipient microbial community. These changes were accompanied by reduced intestinal Claudin-1 expression, increased circulating lipopolysaccharide, resistin, and matrix metalloproteinase-9 concentrations, and decreased circulating primary and secondary bile acids. By transferring a human disease-associated microbiota into a controlled rodent model, this study provides experimental support for the proposition that microbial dysbiosis can contribute to gestational metabolic dysfunction through barrier impairment, mild systemic inflammation, bile acid disruption, and endocrine signaling.

### 7.3 Reproductive health and causal validation

The relationship between the gut microbiome and reproductive health was addressed by Qin et al., who combined bidirectional Mendelian randomization with validation in a rat model of primary dysmenorrhea. The genetic analysis supported a causal direction from dysmenorrhea liability toward alterations in selected microbial taxa, whereas robust evidence that the microbiota caused dysmenorrhea was not identified. In the animal model, both ibuprofen and Wenjing Zhitong Decoction reduced pain-related outcomes but generated distinct microbial profiles. Ibuprofen was associated with increased *Staphylococcus*, whereas the traditional formulation enriched *Bifidobacterium*; *Blautia* was also associated with reduced pain-related indicators. The study is methodologically important because it triangulates human genetic evidence with experimental intervention data while avoiding an unsupported assumption that every disease-associated microbial alteration is a primary causal driver.

Collectively, these studies extend the microbiome concept from intestinal community composition to communication among oral, hepatic, metabolic, endocrine, and reproductive systems. They also illustrate several levels of mechanistic evidence, ranging from taxon–metabolite correlations to microbiota transplantation, microbial depletion, administration of candidate commensals, and integration with human genetic analyses. Across these models, microbial metabolites, intestinal permeability, inflammatory signaling, and host metabolic pathways repeatedly emerge as mediators of interorgan communication. However, the direction of causality remains context dependent: the microbiome may initiate a systemic phenotype, mediate the effects of an exposure, or undergo secondary alteration as a consequence of disease.

## 8 Methodological advances and mechanistic interpretation

The studies collected in this Research Topic illustrate the rapid methodological evolution of microbiome-focused therapeutic research, moving from descriptive characterization of microbial changes toward increasingly integrated approaches designed to establish biological mechanisms and causal relationships. Across disease models and therapeutic strategies, a combination of experimental designs, multi-omics analyses, microbial manipulation approaches, and molecular pathway validation has strengthened interpretation of how treatments interact with the gut microbiome and how microbial changes contribute to therapeutic outcomes.

An important methodological advance is the increasing recognition of biological variables that influence microbiome-host interactions. Sex-specific experimental designs, exemplified by the empagliflozin study, demonstrate the importance of considering sex as a determinant of therapeutic response, metabolic regulation, and microbial composition. Similarly, pharmacokinetic approaches, such as those applied in the losartan study, provide essential information regarding how microbiome alterations may influence drug exposure, metabolism, and systemic efficacy. These approaches are particularly relevant for understanding bidirectional drug–microbiome interactions, where therapeutic compounds may both shape microbial communities and be modified by microbial activity.

A major trend across the Research Topic is the integration of microbiome profiling with metabolomics. Studies involving Jinfu'an Decoction, Jiawei Buyang Huanwu Decoction, electroacupuncture, Zingiber mioga, and multiple intestinal disease models demonstrate that microbial composition alone is insufficient to explain biological effects. Instead, functional interpretation increasingly relies on identifying microbiota-derived metabolites and metabolic pathways that connect microbial alterations with host physiology. This approach has revealed recurrent involvement of bioactive molecules, including short-chain fatty acids such as butyrate and propionate, lipid mediators such as EPA, bile-acid derivatives, TMAO, and tryptophan-derived metabolites.

More direct causal approaches have emerged through microbial-transfer experiments and defined microbial interventions. Fecal microbiota transplantation studies, including FMT combined with andrographolide and microbial restoration approaches in colitis models, allow evaluation of whether therapeutic effects can be transferred through ecosystem-level microbial changes. In parallel, administration of specific bacterial candidates, including *Lactobacillus, Parabacteroides, Faecalibacterium*, and *Bifidobacterium* strains, provides greater resolution regarding strain-associated functions. Defined metabolite supplementation further narrows mechanistic interpretation by separating microbial organisms from their bioactive products.

Pathway-level validation has become essential for connecting microbiome findings with established biological mechanisms. Across the Research Topic, therapeutic effects have been linked to signaling pathways including AhR-mediated immune regulation, TLR4/NF-κB inflammatory activation, Nrf2/Keap1 antioxidant responses, H3K9ac/BDNF/TrkB neurotrophic regulation, YAP1/FXR-associated metabolic signaling, and immune-cell balance involving regulatory and inflammatory populations. Such mechanistic confirmation strengthens the interpretation that microbiome alterations represent functional drivers rather than merely secondary consequences of disease improvement.

An emerging strategy for causal triangulation is the combination of computational approaches with experimental validation, as demonstrated by Mendelian randomization followed by *in vivo* investigation in dysmenorrhea research. This integration of population-level genetic inference with animal experimentation provides a framework for identifying potential causal relationships between microbial factors and disease phenotypes.

Despite these advances, several challenges remain across the field. Many studies continue to rely predominantly on 16S rRNA sequencing, which provides limited strain-level and functional resolution. Differences in animal strains, dietary conditions, housing environments, and treatment duration can also contribute to variability between studies. Furthermore, distinguishing microbiome-mediated therapeutic effects from parallel recovery processes remains difficult. Wider application of germ-free, gnotobiotic, and humanized models, together with greater consideration of sex as a biological variable, will be essential for improving mechanistic precision and translational relevance. Overall, the methodological progression represented in this Research Topic highlights a transition from association-based microbiome research toward experimentally validated, mechanism-driven research.

## 9 Future directions

The 46 contributions included in this Research Topic collectively demonstrate that the gut microbiome represents a dynamic therapeutic interface connecting pharmacological treatments, traditional medicines, microbial therapeutics, and host physiology. However, the next phase of microbiome-based medicine will require a transition from identifying associations toward establishing causality and clinical relevance. Future studies should increasingly incorporate experimental designs capable of determining whether microbial changes are necessary mediators of therapeutic effects or secondary consequences of disease improvement. Gnotobiotic and targeted-colonization models, carefully controlled fecal microbiota transplantation experiments, defined microbial consortia, metabolite supplementation approaches, and receptor- or pathway-specific inhibition strategies will be essential for resolving causal relationships between microorganisms, microbial products, and host responses.

Improving functional resolution will also be a central priority. Although 16S rRNA sequencing has substantially advanced understanding of microbial community changes, future investigations should integrate shotgun metagenomics, metatranscriptomics, metabolomics, and spatial or mucosal microbiome analyses to better define microbial functions, metabolic activity, and interactions with specific intestinal niches. Such approaches will be particularly important for identifying the active microbial pathways responsible for therapeutic efficacy and for distinguishing beneficial organisms from broader community-level changes.

Greater methodological standardization across animal studies will be necessary to improve reproducibility and facilitate comparison across investigations. Detailed reporting of dietary composition, animal sex, genetic background, age, housing conditions, disease induction protocols, treatment duration, sampling procedures, and sequencing workflows should become routine. These variables can substantially influence microbiome composition and may contribute to differences in therapeutic outcomes between laboratories.

An important emerging field is pharmacomicrobiomics, which requires systematic investigation of how microorganisms influence drug bioavailability, metabolism, efficacy, and toxicity. Future studies should examine not only how therapies modify the microbiome but also how microbial enzymes and metabolites alter the pharmacological behavior of conventional drugs, natural compounds, and combination therapies.

The biological heterogeneity of microbiome-mediated responses also requires greater attention. Sex, age, disease severity, genetic background, and baseline microbiome configuration may determine whether an individual responds positively to a given intervention. Identifying these sources of variability will be essential for developing personalized microbiota-based strategies.

Finally, translation from experimental models to clinical application will require validation using human biospecimens, intestinal organoid systems, *ex vivo* microbial–host platforms and appropriately designed clinical trials. Traditional medicines and live biotherapeutics will particularly benefit from improved characterization of active constituents, microbial strains, dose–response relationships, quality control measures, and manufacturing reproducibility. By integrating mechanistic rigor with translational standardization, future microbiome research can move toward precision therapies that harness microbial ecosystems as reliable components of disease prevention and treatment.

## 10 Concluding remarks

The contributions assembled in this Research Topic establish the gut microbiome as an active participant in therapeutic responses rather than a passive consequence of disease or treatment. Across diverse experimental models, conventional pharmacological agents have been shown to influence microbial communities, while microbial organisms and their metabolites can modify drug disposition, efficacy, toxicity, and systemic physiological effects. Traditional medicines, natural products, and complementary interventions frequently converge on shared biological processes involving microbial metabolites, intestinal barrier integrity, inflammatory regulation, and oxidative stress responses.

At the same time, increasingly direct microbiota-targeted strategies, including fecal microbiota transplantation, probiotics, defined commensal organisms, and microbial metabolites, demonstrate the potential of manipulating microbial ecosystems as therapeutic tools. Gastrointestinal studies consistently highlight interactions among microbiota, epithelial barriers, and immune pathways, while extraintestinal models extend these concepts to neurological, hepatic, pulmonary, renal, cardiovascular, retinal, endocrine, and reproductive systems.

Rodent models remain indispensable for defining these complex relationships because they enable controlled microbial manipulation, mechanistic investigation, and integrated analysis of host responses. Moving forward, rigorous causal experimentation, improved methodological standardization, and validation in human systems will be essential. Collectively, this body of work supports a transition from viewing microbiome alterations as secondary effects of therapy toward recognizing the gut microbiome as a critical mediator of therapeutic efficacy, toxicity, and systemic responses.

